# Trends in Genotypic HIV-1 Antiretroviral Resistance between 2006 and 2012 in South African Patients Receiving First- and Second-Line Antiretroviral Treatment Regimens

**DOI:** 10.1371/journal.pone.0067188

**Published:** 2013-06-26

**Authors:** Gert U. Van Zyl, Tommy F. Liu, Mathilda Claassen, Susan Engelbrecht, Tulio de Oliveira, Wolfgang Preiser, Natasha T. Wood, Simon Travers, Robert W. Shafer

**Affiliations:** 1 National Health Laboratory Service, Tygerberg, Coastal Branch, South Africa; 2 Division of Medical Virology, Stellenbosch University, Faculty of Medicine and Health Sciences, Parow, South Africa; 3 Center for AIDS Research, Division of Infectious Diseases, Stanford University Medical Center, Stanford, California, United States of America; 4 Africa Centre for Health and Population Studies, University of KwaZulu-Natal, Mtubatuba, South Africa; 5 South African National Bioinformatics Institute (SANBI), University of the Western Cape, Bellville, South Africa; Rush University, United States of America

## Abstract

**Objectives:**

South Africa’s national antiretroviral (ARV) treatment program expanded in 2010 to include the nucleoside reverse transcriptase (RT) inhibitors (NRTI) tenofovir (TDF) for adults and abacavir (ABC) for children. We investigated the associated changes in genotypic drug resistance patterns in patients with first-line ARV treatment failure since the introduction of these drugs, and protease inhibitor (PI) resistance patterns in patients who received ritonavir-boosted lopinavir (LPV/r)-containing therapy.

**Methods:**

We analysed ARV treatment histories and HIV-1 RT and protease mutations in plasma samples submitted to the Tygerberg Academic Hospital National Health Service Laboratory.

**Results:**

Between 2006 and 2012, 1,667 plasma samples from 1,416 ARV-treated patients, including 588 children and infants, were submitted for genotypic resistance testing. Compared with 720 recipients of a d4T or AZT-containing first-line regimen, the 153 recipients of a TDF-containing first-line regimen were more likely to have the RT mutations K65R (46% vs 4.0%; p<0.001), Y115F (10% vs. 0.6%; p<0.001), L74VI (8.5% vs. 1.8%; p<0.001), and K70EGQ (7.8% vs. 0.4%) and recipients of an ABC-containing first-line regimen were more likely to have K65R (17% vs 4.0%; p<0.001), Y115F (30% vs 0.6%; p<0.001), and L74VI (56% vs 1.8%; p<0.001). Among the 490 LPV/r recipients, 55 (11%) had ≥1 LPV-resistance mutations including 45 (9.6%) with intermediate or high-level LPV resistance. Low (20 patients) and intermediate (3 patients) darunavir (DRV) cross resistance was present in 23 (4.6%) patients.

**Conclusions:**

Among patients experiencing virological failure on a first-line regimen containing two NRTI plus one NNRTI, the use of TDF in adults and ABC in children was associated with an increase in four major non- thymidine analogue mutations. In a minority of patients, LPV/r-use was associated with intermediate or high-level LPV resistance with predominantly low-level DRV cross-resistance.

## Introduction

The South African National Government began providing antiretroviral (ARV) therapy to the public sector in 2004. Until 2009, standard first-line regimens were stavudine (d4T) plus lamivudine (3TC) combined with a third agent: a non-nucleoside reverse transcriptase (RT) inhibitor (NNRTI) in adults and older children or ritonavir-boosted lopinavir (LPV/r) in young children who had received nevirapine (NVP) for prevention of mother-to-child transmission (PMTCT). In 2010, when the South African guidelines were aligned to updated World Health Organization guidelines, D4T was replaced by tenofovir disoproxil fumarate (TDF) in adults and older children and abacavir (ABC) in younger children, respectively. Adults beginning ARV treatment increasingly received TDF rather than d4T for first-line therapy and children increasingly received ABC rather than d4T [1], [2]. The 2004 and 2010 antiretroviral therapy guidelines for adults and children are summarised in Table 1. Although there are ample published data on antiretroviral resistance outcomes of D4T-based regimens in non-subtype B HIV-1 populations, data on the resistance patterns after failure of TDF and ABC-based regimens are limited [3]. Here we examine the effect of ARV usage changes on the patterns of genotypic resistance mutations and their implications for ARV cross-resistance in patients with ARV treatment failure, in a population where HIV-1 subtype C predominates.

**Table 1:** South African National Antiretroviral Therapy Guidelines 2004 and 2010.

Guideline date	2004	2010
**Adults and Adolescents**		
First-line therapy	1D4T, 3TC, ^2^EFV/NVP	Newly initiated patients: ^3^TDF, 3TC/FTC, ^2^EFV/NVP
Definition of virologic failure	Repeated HIV-1 RNA load >5000 copies/ml	Repeated HIV-1 RNA load >1000 copies/ml
Second-line therapy	AZT, DDI, LPV/r	AZT, 3TC, LPV/r (or TDF, 3TC, LPV/r in case of failure of a D4T or AZT containing regimen)
**Children ≤3 years ≤3 kg**		
First-line therapy	D4T, 3TC, LPV/r	ABC, 3TC, LPV/r
Definition of virologic failure	Rebound of HIV-1 RNA load to baseline	Repeated HIV-1 RNA load >1000 copies/ml
Second-line therapy	AZT, DDI, NVP	^4^Refer for expert opinion
**Children >3 years or >10 kg**		
First-line therapy	D4T, 3TC, EFV	ABC, 3TC, EFV
Definition of virologic failure	Rebound of HIV-1 RNA load to baseline	Repeated HIV-1 RNA load >1000 copies/ml
Second-line therapy	AZT, DDI, LPV/r	AZT,DDI, LPV/r

1 D4T could be substituted for AZT in case of toxicity; EFV or NVP chosen dependent on pregnancy risk, EFV chosen when patients receive concurrent rifampicin for tuberculosis. Over time a gradual move to prefer EFV as data suggest that risk to foetus is small. 3TDF replaced by AZT if contra-indicated (e.g. kidney disease). 4Based on data that most children with virologic failuire of a LPV/r first-line regimen have inadequate adherence and no LPV associated resistance, blanket switching is not indicated.

Patients who were still on D4T by the time of the 2010 regimen guidelines could remain on D4T if they did not experience toxicity. However, practically the threshold for switching for lypodystrophy or other side effects is generally low.

## Methods

### Study Population

Since 2006, the National Health Laboratory Service (NHLS) virology laboratory at Tygerberg Academic Hospital has provided ARV genotypic resistance testing for public sector clinics in the Western Cape, Gauteng and Eastern Cape provinces, as allowed by individual clinic or hospital budgets or external funding. Samples received between 2006 and 2012 from children (age below 15 years) and adults with virological failure on a standard first- or second-line regimen for whom demographic and ARV treatment information were provided by their physicians were included in the study. The Stellenbosch University Health Research Ethics Committee approved the study, reference number: N11/09/274.

The treatment histories provided by physicians were assessed in light of the contemporaneous ARV treatment policies in South Africa. Until 2010, the standard adult first-line regimens were d4T combined with 3TC plus either NVP or efavirenz (EFV). Zidovudine (AZT) was often used as a substitute for d4T in case of toxicity. Children younger than three years were generally treated with a boosted lopinavir (LPV/r)-containing first-line regimen under the assumption that a high proportion had been infected with a virus exposed to NNRTI selection pressure as a result of maternal NVP therapy. Initially standard second-line therapy for adults consisted of AZT combined with didanosine (DDI) and LPV/r [4]. Starting in 2010, second-line therapy consisted of 3TC and LPV/r combined with either TDF or AZT, depending on whether patients had failed a thymidine analogue (D4T or AZT)-containing or a TDF-containing first-line regimen. Second-line therapy for children who had started on LPV/r initially included NVP or EFV in combination with two NRTIs. However, according to the 2010 revised guidelines, expert advice had to be obtained for children experiencing failure of a first-line LPV/r regimen, because such failure is mostly due to poor adherence rather than resistance. In some patients, a second-line regimen included three rather than two NRTIs. In adults and children receiving rifampicin concurrently with LPV/r, additional ritonavir was often added, according to therapy guidelines, to increase LPV levels.

In accordance with the revised recommendations, TDF or ABC replaced d4T in a number of patients, who experienced adverse effects, but this was not always noted; therefore a subset of patients recorded as receiving TDF or ABC in combination with 3TC and either NVP or EFV may have previously received d4T.

Laboratory monitoring and detection of failure: The recommended frequency of viral load testing and CD4 testing after initiation of ARV therapy, was six monthly, until the testing frequency was reduced, in 2010, to testing after six months, 12 months and then annually. The definition of virologic failure in adults and adolescents, according to the original South African National Guidelines was repeated HIV viral load measures above 5000 copies/ml. Virological failure in children was defined as rebound of viral load to baseline, and switching to a second-line paediatric regimen was otherwise based on clinical and immunological failure [4]. The definition of virological failure was amended in the 2010 guidelines (Table 1), with virologic failure defined as repeated HIV viral load measures above 1000 copies/ml [1], [2]. However, many clinicians switched patients at a threshold of 1000 copies/ml prior to the publication of the 2010 revised guidelines, based on interim communications, localised guidelines and observational studies that detected resistance at lower viral loads.

### Genotypic Resistance Testing

Standard dideoxynucleotide terminator sequencing was performed using an in-house protocol [5] that amplifies HIV-1 nucleotide positions 2250 to 4229 (HXB2 numbering), spanning the complete PR gene and RT codons 1 to 262. PCR reactions included reagent blanks and, in addition, after each sequencing run, a phylogenetic tree was drawn using all patient sequences and a positive control, included in the run, to enable us to identify possible contamination. Sequences were analysed using the Stanford University HIV Drug Resistance Database (HIVDB) Sierra Webservice (http://hivdb.stanford.edu/pages/webservices). HIV-1 subtyping was performed using the Rega Subtyping Tool [6]. Mutations were defined as amino acid differences from the wild-type consensus HIV-1 B sequence.

The following non-polymorphic ARV-selected mutations were classified as drug resistance mutations (DRM): (i) the NRTI resistance mutations M41L, A62V, K65RN, D67NG, T69D, T69 insertions, T69 deletion, K70REGQ, L74VI, V75MT, F77L, Y115F, F116Y, Q151M, M184VI, L210W, T215YFSDCIV, and K219QENR; (ii) the NNRTI resistance mutations A98G, L100I, K101EPH, K103NS, V106MA, E138KGQ, V179DEFT, Y181CIV, Y188LCH, G190ASEQ, H221Y, P225H, F227LC, M230L, and K238T; and (iii) the PI resistance mutations L10F, V11I, L23I, L24I, D30N, L33F, M46IL, I47VA, G48VM, I50V, I54VMLATS, G73STCA, T74P, L76V, V82ATFSCML, I84V, N88DS, L89V, L90M.

Different RT mutations at the same residue were pooled, including the NRTI-resistance mutations D67NG, K70EGQ, L74VI, M184VI, T215YF, K219QE and the NNRTI-resistance mutations K101EH, K103NS, Y188LCH, and G190ASEQ. Thymidine analogue mutations (TAMs) were defined as M41L, D67NG, K70R, L210W, T215YF, and K219QE. The Q151M complex of mutations was defined as Q151M alone or in combination with one or more of the following mutations: A62V, V75I, F77L, and F116Y. Sequences that terminated between positions 219 and 229 were included in the analysis of NRTI mutation frequency but not in the analysis of NNRTI mutation frequency. The following protease mutations were considered LPV/r-resistance mutations: L10F, L24I, V32I, L33F, M46IL, I47A, I50V, I54MLV, L76V, V82ATSFMC, I84V, L89V, L90M.

When more than one sample was received while a patient received a particular regimen, the sample containing the most drug resistance was chosen, which equalled the cumulative resistance in almost all cases.

### Statistical Analysis

We used the Fisher Exact statistic to compare the proportions of NRTI- and NNRTI-resistance proportions in patients receiving different first-line regimens. Although no explicit correction for multiple comparisons was made, only those differences with a p-value <0.01 were noted.

## Results

### ARV Treatment Regimens

Overall, 1,515 patients had samples submitted for HIV-1 genotypic resistance testing between May 2006 and July 2012. Ninety-four percent (1,416 of 1,515) of patients had a physician-provided ARV treatment history consistent with a standard first- or second-line regimen. Two hundred patients had more than one sample submitted for genotypic resistance testing, including 147 patients who had samples obtained while on different ARV treatment regimens.

Table 2 shows the demographics and ARV treatment histories of these 1,416 patients. Forty-four percent of patients were 15 years or younger at the time of their first sample; 58% were female. The most commonly received NRTI combinations were d4T/3TC (47% of patients), TDF/3TC (13%), AZT/3TC (10%), AZT/DDI (8%), and ABC/3TC (6%). Fifty seven percent of patients had received EFV and seven percent had received NVP. LPV/r was received by 35% of patients including six percent of patients who received a non-standard regimen combining LPV/r and EFV. Ten percent of patients had received a regimen with three NRTIs (the largest number of these combined with an NNRTI).

**Table 2:** Demographic and Antiretroviral Treatments of 1,416 Patients Undergoing HIV-1 Genotypic Resistance Testing, 2006–2012.

		Number	Percent
Gender	Female	821	58
Age*	≤5	210	15
	6 to 10	211	15
	11 to 15	200	14
	16 to 20	62	4
	21 to 30	134	10
	31 to 40	331	23
	41 to 50	201	14
	≥51	67	5
Year of sample*	2006	29	2
	2007	127	9
	2008	119	8
	2009	228	16
	2010	312	22
	2011	396	28
	2012	205	15
Number of samples per patient	1	1216	86
	2	154	11
	≥3	46	3
NRTIs	d4T/3TC	664	47
	TDF/3TC	186	13
	AZT/3TC	138	10
	AZT/DDI	122	8
	ABC/3TC	82	6
	3 NRTIs	140	10
	Misc	84	6
NNRTI/PIs†	EFV	810	57
	LPV/r	413	29
	NVP	94	7
	EFV, LPV/r	86	6

Footnote: *For patients with more than one sample, the age of the patient at the time of the first sample and the year and treatment of the last sample were used.

† The patients receiving EFV and LPV/r included those receiving these ARVs as part of separate regimens and those receiving these as part of salvage therapy.

Misc: Miscellaneous refers to other (rare) NRTI combinations.

TDF use in adults increased from 3% between 2006 and 2009 to 37% between 2010 and 2012 and ABC use from 2% to 6%. In children, ABC use increased from 13% between 2006 and 2009 to 29% between 2010 and 2012. The increase in TDF and ABC use was associated with a concurrent decrease in d4T, DDI and AZT use. Overall, in both age groups, d4T, AZT, and DDI had been used in 65%, 32%, and 20% of patients between 2006 and 2009 and in 45%, 26%, and 14% of patients between 2010 and 2012.

Among the 569 adults receiving first-line regimens 313 (55%) received d4T plus 3TC, 90 (16%) AZT plus 3TC, 153 (27%) TDF plus 3TC, 13 (2%) received ABC plus 3TC. Four hundred and ninety five (87%) received EFV and 74 (13%) NVP. Among the 508 children and infants receiving first-line therapy, 421 (83%) received D4T and 3TC, 65 (13%) ABC plus 3TC and 22 (4%) received AZT plus 3TC. Three hundred and forty one (67%) received EFV, 150 (30%) received LPV/r and 17 (3%) received NVP. Among the 290 adults receiving second-line regimens, the most commonly used regimens included: 126 (43%) receiving AZT plus DDI, 43 (15%) receiving AZT plus 3TC, 37 (13%) receiving TDF plus 3TC/FTC, and 68 (23%) receiving three or more NRTIs, with the rest receiving rare combinations, in each case combined with LPV/r. A wide variety of second-line regimens were used in children and infants, due to the need of expert advice before changing the regimen.

### HIV-1 RT and Protease Sequences

**Figure 1:**
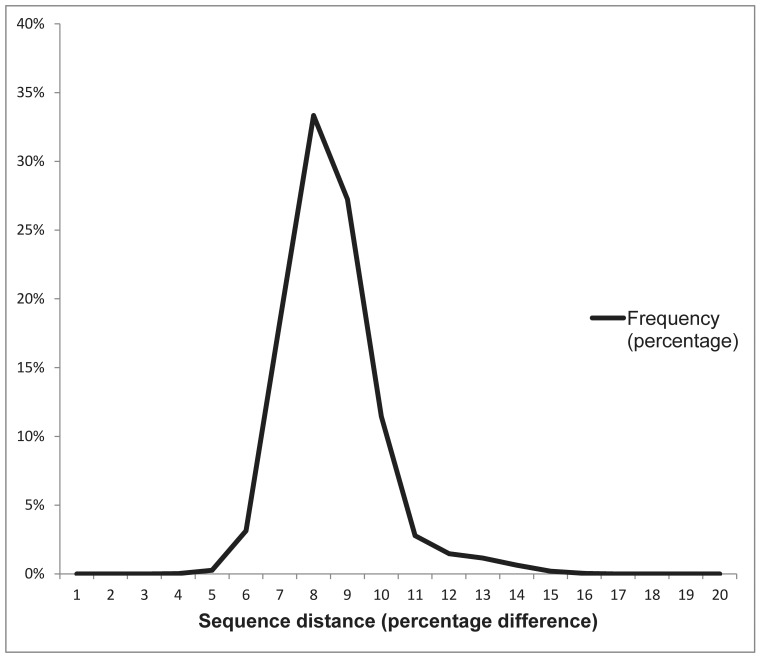
The distribution of uncorrected genetic distance between sequences of different patients. The median uncorrected genetic distance for sequences from different patients was 7.4% (95% range: 5.4% to 11.1%).

**Figure 2:**
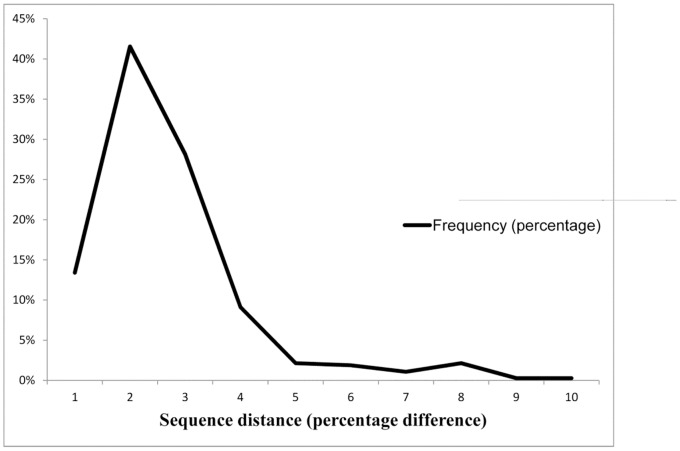
The distribution of uncorrected genetic distance between the sequences from the same patient. The median genetic distance between sequences from the same patient was 1.5% (95% range: 0.1% to 7.5%).

HIV-1 subtype C sequences comprised 98.2% of patient sequences. The remaining virus subtypes included A (0.9%), B (0.1%), BC recombinants (0.6%) and miscellaneous subtypes and recombinant forms (0.2%). The median uncorrected genetic distance (Hamming distance) for sequences from different patients was 7.4% (95% range: 5.4% to 11.1%; Figure 1). Fourty-one patients (2.7% of 1,511) had a virus sequence with a genetic distance <2.0% from another virus sequence; twenty patients (1.3%) had a virus sequence with a genetic distance <1.0% from another virus sequence. The median genetic distance between sequences from the same patient was 1.5% (95% range: 0.1% to 7.5%; Figure 2). The GenBank accession numbers of the sequences are KC422792–KC424425.

### Genotypic ARV-resistance during First-line Dual NRTI/NNRTI Treatment

#### NRTI-resistance mutations

Table 3 shows the proportions of the most common NRTI-resistance mutations in the 927 adults and children receiving a first-line dual NRTI plus NNRTI regimen. Three mutations – K65R, L74VI, and Y115F – occurred in a higher proportion of patients receiving TDF and ABC compared to patients receiving d4T or AZT. K70EG occurred in a higher proportion of patients receiving TDF compared to patients receiving d4T or AZT.

**Table 3:** Nucleoside RT Inhibitor (NRTI) Resistance Mutations: Percent Occurrence in Patients Treated with Dual NRTI plus Nonnucleoside RT Inhibitor (NNRTI) First-Line Antiretroviral (ARV) Regimens.

ARV Regimen		No.*	184†VI	Thymidine Analogue Mutations (TAMs)								Discriminatory Mutations					
NRTIs	NNRTI			41	67	70	210		215	219	69	65	69	70	74	115	151
			(%)	L	NG	R	W		YF	QE	ins	R	del	EQG	VI	F	M
				(%)	(%)	(%)	(%)		(%)	(%)	(%)	(%)	(%)	(%)	(%)	(%)	(%)
***Thymidine analog-based regimens***																	
d4T/3TC	EFV	573	82	1.8	9.6	5.1		0.2	3.5	3.5	0.2	3.3	0.5	0.5	1.9	0.7	0.5
	NVP	42	88.1	9.5	16.7	2.4		2.4	19	4.8	0	0	0	0	0	0	0
AZT/3TC	EFV	76	76.3	7.9	27.6	25		5.3	26.3	19.7	0	1.3	0	0	2.6	0	0
	NVP	29	86.2	0	27.6	27.6		0	13.8	17.2	0	0	0	0	0	0	0
***Totals*** § ***:***		720	81.9^*^	2.8	12.6	7.9		0.8	7.2^*^	5.8	0.1	2.8^**^	0.4	0.4^**^	1.8^**^	0.6^**^	0.4
***Tenofovir (TDF) and abacavir (ABC)-based regimens***																	
TDF/3TC	EFV	133	63.9	0.8	13.5	6	0		3.0	9.8	0	40.6	1.5	8.3	8.3	10.5	1.5
	NVP	20	90	5.0	5.0	0	0		5.0	5.0	0	80	5.0	5	10	10	5.0
ABC/3TC	EFV	54	81.5	1.9	7.4	0	0		0	1.9	0	16.7	0	0	55.6	29.6	1.85
***Totals*** § ***:***		207	71^*^	1.5	11.1	3.9	0		2.4^*^	7.2	0	38.2^**^	1.5	5.8^**^	20.8^**^	15.5^**^	1.9

Footnote: *No.: Number of patients receiving first-line therapy with the ARV regimen indicated in the first two columns.

† Although M184V/I is a discriminatory mutation it is shown separately because it is the single most common mutation.

§ The proportion of individuals receiving a thymidine analog (d4T or AZT) or non-thymidine analog (TDF or ABC) based regimen having the indicated mutation. Mutations for which there were statistically significant differences between these proportions are in bold, *p≤0.01, **p≤0.001.

K65R occurred in 70/153 (45.8%) patients failing ARV and receiving TDF and in 9/54 (16.7%) patients receiving ABC compared with 20/720 (2.8%) patients receiving d4T or AZT (p<0.001 for both TDF and ABC). L74VI occurred in 13/153 (8.5%) patients receiving TDF and 30/54 (55.6%) receiving ABC compared with 1.8% receiving d4T or AZT (p<0.001 for both TDF and ABC). Y115F occurred in 16/153 (10.5%) patients receiving TDF and 16/54 (29.6%) receiving ABC compared with 4/720 (0.6%) patients receiving d4T or AZT (p<0.001; for both TDF and ABC). K70QEG occurred in 12/153 (7.8%) patients receiving TDF compared with 3/720(0.4%) receiving d4T or AZT (p<0.001).

TAMs occurred in a higher proportion of patients receiving an AZT-containing regimen than in patients receiving a TDF- or ABC-containing regimen (39% vs. 18%; p<0.001). However, there was no difference in the proportion of patients with TAMs between those receiving a d4T-containing regimen compared with those receiving a TDF- or ABC-containing regimen (16% vs. 18%). M184V/I (82.8% vs. 72%; p = 0.006) occurred in a somewhat higher proportion of patients receiving a d4T- or AZT-based first-line regimen than an ABC- or TDF-based regimen.

K65R occurred in a higher proportion of patients receiving TDF plus 3TC plus NVP than TDF plus 3TC plus EFV (16/20; 80% vs. 54/133; p = 0.001) but there were otherwise no significant differences in the proportions of NRTI-resistance mutations between the 91 patients receiving an NVP-containing regimen compared with the 836 receiving an EFV-containing regimen.

Additional NRTI resistance mutations not shown in Table 3 included (i) A62V, which occurred more commonly in patients receiving TDF (19/153; 12.4%) than in those receiving AZT, d4T, or ABC (36/774, 4.7%; p<0.001); and among TDF-recipients, A62V occurred more commonly in samples with K65R plus M184V (14/39, 35.9%) than in samples with M184V alone (4/50, 8%; p<0.001), K65R alone (1/31, 3.2%; p<0.001), or neither K65R nor M184V (0/33, 0%; p<0.001). (ii) K65N, which occurred in one patient receiving TDF; (iii) T69D, V75M, and V75T, which occurred in 1.3%, 2.5%, and 0.3% respectively; (iv) T215I occurred in 1.4% of patients; and (v) K219R and K219N, occurred in 1.6% and 0.9% respectively.

An analysis of the complete set of RT sequences, identified two novel, possibly subtype C-associated, NRTI mutations. T165L, a nonpolymorphic mutation previously reported to be associated with NRTI therapy occurred in 1.6% (22) of patients, a proportion similar to the 1.3% found in the ∼3,600 RTI-experienced subtype C-infected patients in HIVDB but significantly higher than the 0.4% found in the ∼25,000 RTI-experienced non-subtype C-infected patients in HIVDB. S68N occurred in 1.4% (20) of patients, a proportion higher than the 0.1% and 0.3% previously found in the subtype C and non-subtype C RTI-experienced patients in HIVDB. Of note, 18 of the 20 patients with this mutation also had K65R.

#### NNRTI-resistance mutations

Table 4 shows the proportions of NNRTI-resistance mutations among the 887 adults and children receiving a first-line dual NRTI/NNRTI containing regimen. A higher proportion of patients receiving EFV (37% of 801) had viruses with V106M compared with those receiving NVP (12% of 86; p<0.001). A higher proportion of patients receiving NVP (41% of 86) had viruses with Y181C compared with those receiving EFV (5% of 801; p<0.001). Among the patients receiving EFV, L100I occurred in a higher proportion of the 53 patients receiving ABC/3TC (23%) compared with the remaining 748 patients (3.1%; p<0.001) and Y181C occurred in a higher proportion of the 127 patients receiving TDF/3TC (18%) compared with the remaining 674 patients (2.5%; p<0.001). Y188C, a mutation previously reported in 0.1% of non-subtype C sequences occurred in 1.5% (21) patients in this study. In 19 of 21 patients, Y188C occurred in combination with V106M.

**Table 4:** Nonnucleoside RT Inhibitor (NNRTI) Resistance Mutations: Percent Occurrence in Patients Treated with Dual nucleoside RT inhibitor (NRTI) plus NNRTI First Line Antiretroviral (ARV) Regimens.

ARV Regimen		No.*	100	101	101	103	106	138	181	188	190	230
NNRTI	NRTI		L	P	EH	NS	M	K	C	LCH	ASEQ	L
			(%)	(%)	(%)	(%)	(%)	(%)	(%)	(%)	(%)	(%)
***Efavirenz (EFV)-containing regimens***												
EFV	d4T/3TC	548	3.3	1.5	11.7	56.4	35.2	1.1	2.4	9.9	15.1	6.9
	AZT/3TC	73	1.4	1.4	5.5	49.3	27.4	2.7	1.4	12.3	9.6	9.6
	TDF/3TC	127	3.1	1.6	15.7	32.3	48.8	0	18.1	9.4	21.3	4.7
	ABC/3TC	53	22.6	1.9	11.3	56.6	37.7	3.8	5.7	7.5	11.3	1.9
***Totals*** † ***:***		801	4.4	1.5	11.7	51.9	36.8**	1.2	5.0**	9.9	15.4	6.5
***Nevirapine (NVP)-containing regimens***												
NVP	d4T/3TC	40	0	2.5	22.5	52.5	15	0	37.5	7.5	10	5
	AZT/3TC	27	0	0	25.9	25.9	14.8	0	25.9	3.7	29.6	11.1
	TDF/3TC	19	0	5.3	10.5	36.8	0	0	68.4	0	31.6	10.5
***Totals*** † ***:***		86	0	2.3	20.9	40.7	11.6**	0	40.7**	4.7	20.9	8.1

Footnote: *No.: Number of patients receiving first-line therapy with the ARV regimen indicated in the first two columns.

† The proportion of individuals treated with NVP or EFV. Mutations for which there were statistically significant differences according to the NNRTI received: **p≤0.001.

Fewer sequences were included in table 4 than in table 3 as sequences that terminated between positions 219 and 229 were excluded from the numerator and denominator for NNRTI mutation statistics.

Additional NNRTI-resistance mutations not shown in Table 4 included (i) A98G, which occurred in 3.3% of NNRTI-treated patients; (ii) V106A, which occurred in 0.6% of NNRTI-treated patients; (iii) E138G/Q, which occurred in 0.9% and 0.9% of patients, respectively; (iv) V179D/E/T/F, which occurred in 8.5%, 0.7%, 0.5%, and 0% of NNRTI-treated patients respectively; (v) Y181I/V, which occurred in one and no patient, respectively; (vi) H221Y, which occurred in 5.9% of EFV-treated and 9.3% of NVP-treated patients (p<0.001); (vii) P225H, which occurred in 13.6% of EFV-treated and 5.8% of NVP-treated patients (p = 0.01); and (viii) F227C, which occurred in three patients; and (ix) K238T, which occurred in 2.3% of NNRTI-treated patients.

### PI-resistance Mutations in Patients Receiving LPV/r

Of the 490 patients who received an LPV/r-containing regimen, 55 (11.2%) had plasma virus samples with one or more LPV-resistance mutations. These 55 plasma virus samples comprised 36 distinct patterns of LPV-resistance mutations (Table 5). Overall, the genotype predicted intermediate or high-level LPV resistance in 45 patients, intermediate or high-level ATV resistance in 36 and DRV resistance in 23 (intermediate (n = 3) or low-level (n = 20)) patients. Because 18 of the 48 patients with LPV resistance also had NRTI and NNRTI resistance, the overall proportion of patients with three-class resistance was 1.3% (18/1416).

**Table 5:** Protease Inhibitor (PI)-Resistance Mutation Patterns in Viruses From Patients Receiving Lopinavir/r and their Predicted Effect on PI Cross Resistance*.

No. Mut	Mutation List†	NumPts	LPV§	ATV§	DRV§
1	L10F	1	5	0	0
	L33F	1	5	5	5
	M46L	1	10	10	0
	I47A¶	1	**60**	**70** ¶	10
	I54V	1	10	15	0
	L76V	1	**30**	0	20
	V82A	1	25	15	0
	I84V	1	15	**45**	10
	L90M	1	10	20	0
2	I54V, V82A	6	**35**	**35**	0
	L10F, V82A	4	**30**	15	0
	M46I, L76V	2	**50**	7.5	20
	I54V, I84V	1	**25**	**55**	10
	M46I, V82A	1	**35**	**30**	0
	M46I, I50V	1	**30**	10	20
	V32I, I47A	1	**>60**	20	**30**
3	M46I, I54V, V82A	3	**55**	**50**	0
	I54V, L76V, V82A	2	**>60**	**45.5**	20
	L24I, V32I, I47A	1	**>60**	25	**30**
	L10F, L76V, V82A	1	**60**	15	20
4	M46I, I54V, L76V, V82A	3	**>60**	**>60**	20
	M46I, I50V, I54V, V82A¶	2	**>60**	>60¶	20
	L10F, M46I, I54V, V82A	2	**>60**	60*	20
	L10F, I54V, I84V, L89V¶	1	**35**	**>60** ¶	15
	L10F, L33F, I54V, V82A	1	**55**	**45**	5
	L10F, L24I, I54V, V82A	1	**>60**	**45**	0
5	L10F, M46I, I54V, L76V, V82A	4	**>60**	**55**	20
	L10F, M46L, I54V, L76V, V82A	1	**>60**	**55**	20
	L10F, M46I, I54V, V82A, I84V	1	**50**	**>60**	10
	L10F, M46I, I54V, L76V, I84V	1	**>60**	**60**	**30**
	L10F, L24I, L33F, I54V, V82A	1	**>60**	**55**	5
	L10F, L33F, I54V, L76V, V82A	1	**>60**	**40**	25
6	L10F, L24I, L33F, M46I, I54V, V82A	1	**>60**	**>60**	5
	L10F, L24I, L33F, M46L, I54V, V82A	1	**>60**	**>60**	5
	L10F, L33F, M46I, I54V, V82A, L90M	1	**>60**	**>60**	5
	L10F, L33F, M46I, I50V, I54V, V82A	1	**>60**	**>60**	25
7	L10F, L24I, L33F, M46I, I54V, L76V, V82A	1	**>60**	**>60**	25

Footnote: *36 patterns of PI-resistance mutations from 55 patients.

† PI-resistance mutations included L10F, L24I, L33F, V32I, M46I/L, I50V, I54V, L76V, V82A, I84V, L89V, and L90M. (D30N, I47V, G48V, I50L, I54L/M/T/A/S, and V82T/S/F did not occur in this dataset). V82M and V82C occurred in 2 patients and were represented by V82A. The accessory mutations L10I/V and A71V/T occurred commonly but are not shown. The mutations V11I, F53L, G73S, T74P, N83D, and N88S each occurred in 1 to 3 patients and are also not shown.

§ Predicted reduced susceptibility to lopinavir/r (LPV), atazanavir/r (ATV), and darunavir/r (DRV) according to the HIVDB drug-resistance interpretation system. Scores ≥60 indicate high-level resistance; scores between 30 and 59, intermediate resistance; scores between 15 and 29, low-level resistance.

¶ One of more samples with this pattern of study-defined LPVr mutations had additional PI-resistance mutations that influenced the extent of ATVr cross resistance. For example, the sample with I47A also had the mutation N88S which is associated with high-level ATVr resistance.

## Discussion

In the nine years since the start of the South African National ARV Treatment Program, therapy has evolved in accordance with WHO ARV treatment recommendations. TDF and ABC have increasingly been used in place of d4T and AZT in patients receiving first-line therapy and the number of patients requiring second-line therapy with LPV/r has gradually increased [2]. In this study, we assessed the effect of the expanded use of TDF and ABC on the patterns of NRTI resistance in patients experiencing first-line virological failure and the extent of PI cross-resistance among patients receiving LPV/r.

The data presented in this study substantially increases the number of publicly available sequences, as of April 2013, from ARV-treated subtype C infected patients. The subtype C RT sequences from 1,398 NRTI ± NNRTI-treated patients represents nearly 40% of the ∼3,600 of such patients in HIVDB. The subtype C PR sequences from 486 LPV/r-treated patients more than double the number of all subtype C-infected patients in HIVDB.

Among patients with virological failure on a first-line dual NRTI plus NNRTI regimen, a higher proportion of those who received TDF and/or ABC had the non-TAMs K65R, K70EQG, L74VI, and Y115F compared with those receiving d4T or AZT. M184V, the most common NRTI-resistance mutation, occurred in a significantly but only modestly higher proportion of patients receiving an AZT- or d4T-containing regimen compared with those receiving a TDF- or ABC-containing regimen.

With the exception of infrequent mutations at codons 67 and 219, TAMs rarely occurred in patients receiving ABC plus 3TC- or TDF plus 3TC-containing first-line regimens. The fact that 21% of TDF recipients and 10% of ABC recipients had TAMs is therefore consistent with the substitution of these NRTIs for managing d4T toxicity. Such single drug switches in patients without documented virological suppression is a likely explanation for the higher than expected frequency of TAMs in TDF and ABC recipients.

The high proportion of non-TAMs in patients receiving first-line TDF- and ABC-containing regimens is a striking example of HIV-1′s ability to evolve under different selection pressures. The high proportion of non-TAMs is also of concern in that the number of ARV-resistance mutations developing during virological failure is often related to the duration of failure. Indeed, the risk of K65R was significantly higher in the 20 patients receiving TDF plus 3TC plus NVP compared to the 133 patients receiving TDF plus 3TC plus EFV, a finding consistent with previous reports questioning the efficacy of TDF plus 3TC plus NVP [7], [8].

As our study was a laboratory-based study, we were not able to evaluate the response to therapy with TDF- and ABC-containing regimens. Indeed, patients in this study who were treated with an initial TDF- or ABC-containing regimen or with TDF or ABC as a substitute for d4T may have had more advanced HIV-1 disease. However, a recent retrospective South African study of 585 patients receiving first-line therapy with TDF and 3TC plus an NNRTI was particularly informative [3]. It reported that despite a low prevalence of virological failure of six percent, the median time to presentation for those with virological failure was only six months. Whether rapid failure occurred more commonly among those receiving TDF plus 3TC plus NVP as compared to TDF plus 3TC plus EFV was not indicated. As in our study, M184V, K65R, and Y115F were the most common major NRTI mutations.

The distribution of NNRTI resistance mutations was consistent with previous studies: V106M was significantly more common among patients receiving EFV and Y181C was significantly more common among patients receiving NVP [9], [10], [11], [12]. The statistical associations of Y181C with TDF and of L100I with ABC have not previously been reported. The former association is surprising because Y181C increases TDF susceptibility [13], [14]. The presence of L74V in patients who failed TDF-based regimen was also surprising as L74V increased TDF susceptibility *in vitro* [14], [15].

The 11% prevalence of LPV/r resistance among patients with virological failure on an LPV/r containing regimen is consistent with studies from the U.K. [16] and from South Africa [17]. This finding suggests that in patients with virological failure on an LPV/r-containing regimen, genotypic resistance testing can distinguish those who may respond to improved adherence from those who require a change in therapy. The development of just low level DRV resistance in about one-half of the patients with LPV resistance, or rarely intermediate resistance, suggests that DRV may be a valuable component of a third-line antiretroviral regimen.

In conclusion, changes in HIV treatment practices are greatly influencing the genotypic patterns of ARV resistance and cross-resistance in patients experiencing first-line ARV treatment failure. Indeed a high proportion of patients receiving TDF or ABC had non-TAMs that were uncommonly observed in patients receiving AZT or d4T. Although TDF and ABC are more potent and less toxic HIV-1 inhibitors than d4T and AZT, the impact of their widespread introduction in South Africa requires ongoing monitoring to ensure that the efficacy of first-line therapy is not compromised, and to identify optimal second-line regimens.

Currently over 1.7 million patients are on ARV therapy in South Africa and with the anticipated increased complexity of regimens as therapy history increases, an increased need for resistance testing is expected. Importantly, in order to inform national and international policy makers, genotypic data and its associated treatment regimen should be made available in public databases [18].
